# Introduction of Aedes albopictus into a La Crosse virus--enzootic site in Illinois.

**DOI:** 10.3201/eid0404.980413

**Published:** 1998

**Authors:** U Kitron, J Swanson, M Crandell, P J Sullivan, J Anderson, R Garro, L D Haramis, P R Grimstad

**Affiliations:** College of Veterinary Medicine, University of Illinois, Urbana 61801, USA. ukitron@uiuc.edu

## Abstract

In late summer and fall 1997, Aedes albopictus mosquitoes were found in Peoria, Illinois, a long recognized focus of La Crosse virus transmission. Larvae were found in tires and other artificial containers, biting adults were recovered, and eggs were collected in oviposition traps within a 25-ha area. One chipmunk trapped < 0.25 km from the infested area tested positive for neutralizing antibodies against La Crosse virus.


### Introduction of *Aedes albopictus* into a La Crosse Virus–Enzootic Site in Illinois

**Uriel Kitron,* Jack Swanson,† Michael Crandell,‡ Patrick J. Sullivan,* Justin Anderson,§ Robert Garro,* Linn D. Haramis,¶ and Paul R. Grimstad§**
*University of Illinois, Urbana, Illinois, USA; †Illinois Department of Public Health, Peoria, Illinois, USA; ‡Peoria City/County Health Department, Peoria, Illinois, USA; and §University of Notre Dame, Notre Dame, Indiana, USA; ¶Illinois Department of Public Health, Springfield, Illinois, USA

In late summer and fall 1997, *Aedes albopictus* mosquitoes were found in Peoria, Illinois, a long recognized focus of La Crosse virus transmission. Larvae were found in tires and other artificial containers, biting adults were recovered, and eggs were collected in oviposition traps within a 25-ha area. One chipmunk trapped <0.25 km from the infested area tested positive for neutralizing antibodies against La Crosse virus.

*Aedes albopictus* (Skuse) was probably introduced into the United States in used tires from northern Asia (1) and has spread throughout much of the southern United States, often displacing *Ae. aegypti* (L.) (2,3). Although *Ae. albopictus* mosquitoes have a short flight range (200 m to 600 m), they are readily transported in containers and vehicles (3,4).

In laboratory studies, *Ae. albopictus* was a more efficient vector of La Crosse (LAC) virus than the natural vector, *Ae. triseriatus* (Say) (5), and could readily transmit LAC virus to chipmunks (6). In field and laboratory studies of larval competition in tires and artificial containers, *Ae. albopictus* outnumbered *Ae. triseriatus* (7,8). LAC virus has not been isolated from field-caught *Ae. albopictus* females in an endemic-disease area.

LAC virus infection, the most common cause of pediatric arboviral encephalitis in the United States (9), is endemic in several midwestern and mid-Atlantic states (10). In Illinois, human clinical cases of LAC virus encephalitis are concentrated around Peoria, often near discarded tires and containers (11,12). LAC virus encephalitis is one of the main threats associated with the introduction of *Ae. albopictus* to the midwestern United States (1,2).

Address for correspondence: Uriel Kitron, College of Veterinary Medicine, University of Illinois, 2001 S. Lincoln, Urbana, IL 61801, USA; fax: 217-244-7421; e-mail: u-kitron@uiuc.edu.

### Mosquito Surveillance

Peoria City/County Health Department and Illinois Department of Public Health personnel annually conduct surveillance of container-breeding mosquitoes in Peoria County by searching for and monitoring artificial containers, collecting adult females, and monitoring oviposition activity (13).

During an investigation of a suspected LAC virus encephalitis case, *Ae. albopictus* larvae and adults were collected on August 20, 1997, from approximately 10 tires and a steel box. During annual surveillance in August and September 1997, *Ae. albopictus* larvae and pupae were collected from tires and other containers. Adult mosquitoes were collected near most breeding sites and in several additional locations (Figure). Approximately 160 *Ae. albopictus* and one *Ae. triseriatus* adult females were collected. All artificial containers with mosquito larvae were treated with Abate larvicide. During the first week of October, larvae were also collected from a small boat containing water, and adults were collected in the immediate vicinity.

Nine oviposition traps were placed in the infested site starting August 21, 1997 (Figure). The *Aedes* eggs found in seven traps were hatched, and the larvae were reared to adults. *Ae. albopictus* were identified from four traps, with two traps each yielding more than 100 mosquitoes. *Ae. triseriatus* were identified from

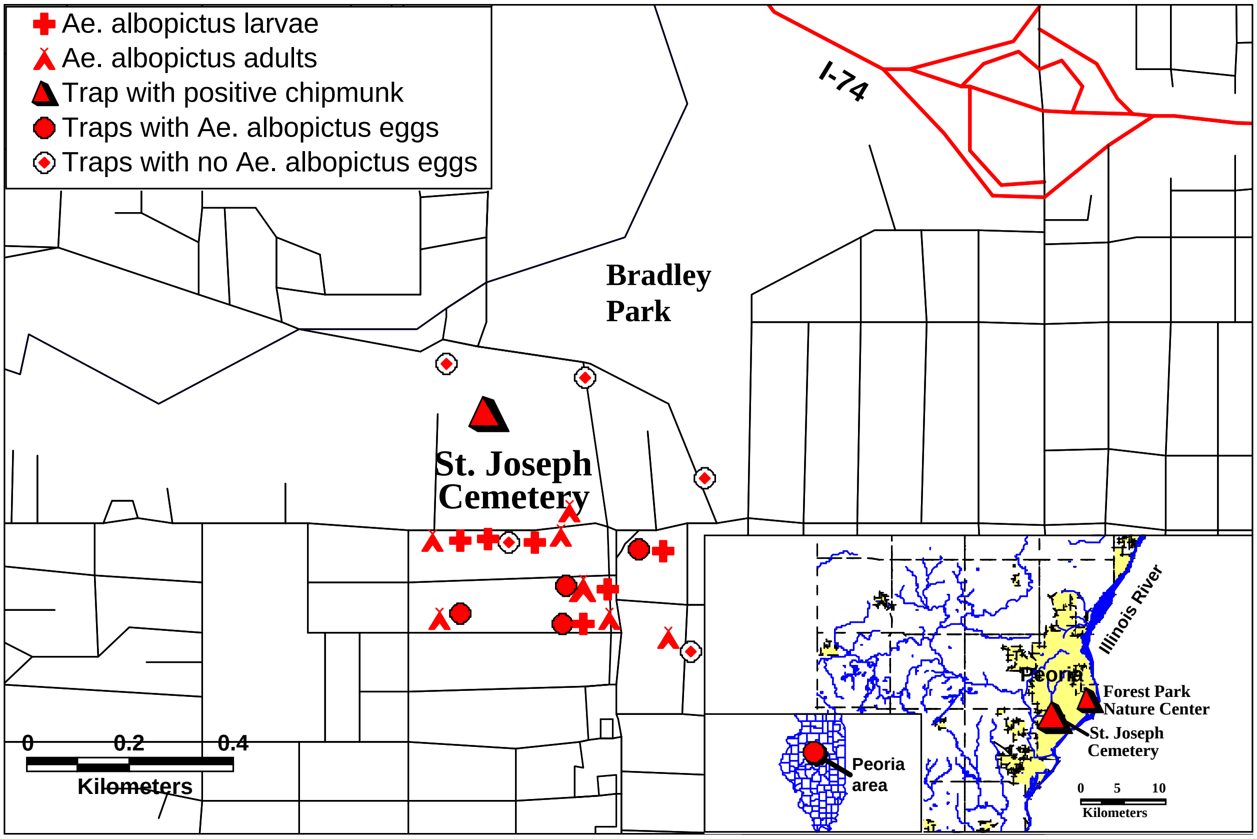

Figure. Locations in Peoria, Illinois, where *Aedes albopictus* larvae and adults were found and where a La Crosse–positive chipmunk was trapped. Location of oviposition traps are shown, including traps where no *Ae. albopictus* eggs were deposited. Insets show location of Peoria and of the study sites.

four traps, with the total numbering less than half of *Ae. albopictus*. No LAC virus was isolated from these field-collected mosquitoes.

### Reservoir Host Trapping and Sampling

Fifteen Tomahawk live traps (model 102/rat: 40.6 x 12.7 x 12.7 cm.; Tomahawk Live Trap Company, Madison, WI) were placed 5 to 15 m apart in an east-west transect in St. Joseph Cemetery, Peoria, Illinois, on September 20, 1997 (Figure). To attract chipmunks and squirrels, traps were baited with sunflower seeds and small pieces of peanut butter sandwiches. Trapped animals were processed and released after recovering from anesthesia. Animal trapping and handling procedures were conducted in accordance with a protocol approved by the Laboratory Animal Care Advisory Committee at the University of Illinois (protocol #V6R131).

In St. Joseph Cemetery, 10 chipmunks (*Tamias striatus*) were caught in 15 traps opened one morning (Figure). This 67% trapping success rate is high compared with the rate at Forest Park Nature Center (Figure), a long-recognized LAC virus–enzootic transmission focus in Peoria (11), where on the same date, chipmunks and squirrels (*Sciurus carolinensis* and *S. niger*) were collected in 15 (30%) of 50 traps.

### LAC Antibody Detection

Blood (0.2 ml) was taken from anesthetized chipmunks by a suborbital sinus puncture behind the right eye with a 100-µl capillary tube (14) and absorbed onto two Nobuto (Toyo Roshi Kaisha, Tokyo, Japan) filter strips, which were tested for antibody at the University of Notre Dame. Neutralizing (N) antibodies against LAC virus were detected by a cell culture assay and the serum-dilution neutralization test (SDNt) (15). Titers, calculated by the method of Reed and Muench (16), were expressed as the highest dilution showing <50% cytopathic effects. Homologous (LAC virus) and heterologous

(Jamestown Canyon, Trivittatus, eastern equine encephalitis, St. Louis encephalitis virus) mouse hyperimmune ascitic fluids served as positive and negative N antibody controls, respectively.

Of the 10 chipmunks trapped in St. Joseph Cemetery, 1 (10%) was positive (titer = 8) for N antibodies against LAC virus. Two (22%) of nine chipmunks trapped on the same date in Forest Park Nature Center were N-antibody positive (titers 4, 16). Low titers may be associated with the differing amounts of sera present in the Nobuto strips (all sera were eluted in the same amount of media and then diluted as if all were equal). Low titers may also be caused by animals having been infected earlier in the season. Because little serum was available, we conducted only SDNt, our most sensitive and specific serologic procedure for detecting California serogroup positives.

### Mapping

The spatial distribution of mosquito collections (adults and larvae), locations and catches of oviposition traps, and locations of chipmunk traps were overlaid on a street map of the city of Peoria (Figure). The LAC virus–seropositive chipmunk caught in St. Joseph Cemetery was trapped approximately 150 m from sites where *Ae. albopictus* eggs, larvae, and adults were collected (Figure). These data were compared with data on human cases (with known addresses) (12); all epidemiologic and entomologic data were stored in a geographic information system (MapInfo GIS, Troy, NY) as part of an ongoing study. The system allows for ready management of georeferenced data, continuous updating of entomologic and epidemiologic data, and production of custom maps.

### Conclusion

The detection of *Ae. albopictus* mosquitoes, coupled with the trapping of an LAC virus–seropositive chipmunk within 150 m of larval and adult *Ae. albopictus* collection sites (well within the flight range of *Ae. albopictus*), is noteworthy. Human cases were reported within 1.5 km of these sites in the 1970s and in 1994. Even within Peoria, the spatial distribution of enzootic foci and human cases is patchy. These findings are the first evidence that *Ae. albopictus* has been introduced into the heart of an urban and suburban area where LAC virus has long been endemic and has caused disease in humans. A focus in rural southeastern Indiana has been described (17) in a highly LAC virus-endemic area of the state (18).

Studies in Indiana and an estimate of the northern limits of the distribution of *Ae. albopictus* (19-21) suggest that *Ae. albopictus* may become entrenched in central Illinois, especially after mild winters. *Ae. albopictus* eggs stored outdoors at the University of Notre Dame in northern Indiana were hatched successfully in late February 1998, following several days of record warm temperatures, and in mid-April after cold weather had passed. In addition, *Ae. albopictus* have long been detected in Indianapolis, Indiana, at a latitude close to that of Peoria, Illinois (M. Sinsko, Indiana State Board of Health, pers. comm.).

The detection of this *Ae. albopictus* focus marks the beginning of a natural experiment to test the ability of *Ae. albopictus* to overwinter in central Illinois, displace *Ae. triseriatus* from artificial and natural containers, and survive intensive control efforts. If this population of *Ae. albopictus* reemerges in 1998, its vertebrate host feeding preference and its ability to act as a vector for enzootic and endemic transmission of LAC virus should also be evaluated.

### Acknowledgment

M. Guerra and M. Lancaster provided fieldwork assistance.

This study was supported by the Used Tire Management Fund, State of Illinois, U.S. Department of Agriculture Animal Health and Disease Funds, a UIUC Research Board award to U. Kitron, and NIH/NIAID grant AI02753 to P. Grimstad.

Uriel Kitron is professor and chair of the Division of Epidemiology and Preventive Medicine in the Department of Veterinary Pathobiology, College of Veterinary Medicine, University of Illinois. He studies the ecology and epidemiology of vector-borne diseases, including malaria, Lyme disease, and mosquito-borne arboviruses. He is particularly interested in landscape ecology, spatial analysis, and risk assessment of infectious diseases.

### References

1. Hawley WA, Reiter P, Copeland RS, Pumpuni CB, Craig GB Jr. *Aedes albopictus* in North America: probable introduction in used tires from northern Asia. Science 1987;236:1114-6.
2. Rai KS. *Aedes albopictus* in the Americas. Annu Rev Entomol 1991;36:459-84.

3. Moore CG, Mitchell CJ. *Aedes albopictus* in the United States: ten-year presence and public health implications. Emerg Infect Dis 1997;3:329-34.
4. Estrada-Franco JG, Craig GB Jr. Biology, disease relationships, and control of *Aedes albopictus*. Pan American Health Organization Technical Paper No. 42; 1995.
5. Grimstad PR, Kobayashi JF, Zhang M, Craig GB Jr. Recently introduced *Aedes albopictus* in the United States: potential vector of La Crosse virus (Bunyaviridae: California serogroup). J Am Mosq Control Assoc 1989;5:422-7.
6. Cully JF Jr, Street TG, Heard PB. Transmission of La Crosse virus by four strains of *Aedes albopictus* to and from the eastern chipmunk (*Tamias striatus*). J Am Mosq Control Assoc 1992;8:237-40.
7. Ho BC, Ewert A, Chew LM. Interspecific competition among *Aedes aegypti, Ae. albopictus* and *Ae. triseriatus* (Diptera: Culicidae): larval development in mixed cultures. J Med Entomol 1989;26:615-23.
8. Novak MG, Higley LG, Christianssen CA, Rowley WA. Evaluating larval competition between *Aedes albopictus* and *A. triseriatus* (Diptera: Culicidae) through replacement series experiments. Environmental Entomology 1993;22:311-8.
9. Calisher CH. Medically important arboviruses of the United States and Canada. Clin Microbiol Rev 1994;7:89-116.
10. Grimstad PR. California group viruses. In: Monath TP, editor. The arboviruses: epidemiology and ecology. Vol II. Boca Raton (FL): CRC Press; 1988. p. 99-136.
11. Clark GG, Pretula HL, Rohrer WH, Harroff RN, Jakubowski T. Persistence of La Crosse virus (California encephalitis serogroup) in north-central Illinois. Am J Trop Med Hyg 1983;32:175-84.
12. Kitron U, Michael J, Swanson J, Haramis L. Spatial analysis of the distribution of La Crosse encephalitis in Illinois, using a geographic information system and local and global spatial statistics. Am J Trop Med Hyg 1997;57:469-75.
13. Moore CG, McLean RG, Mitchell CJ, Nasci RS, Tsai TF, Calisher CH, et al. Guidelines for arbovirus surveillance programs in the United States. Fort Collins (CO): U.S. Department of Health and Human Services; 1993.
14. McLean RG, Ubico SR, Cooksey LM. Experimental infection of the eastern chipmunk (*Tamias striatus*) with the Lyme disease spirochete (*Borrelia burgdorferi*). J Wild Dis 1993;29:527-32.
15. Pantuwatana S, Thompson WH, Watts DM, Hanson RP. Experimental infection of chipmunks and squirrels with La Crosse and trivittatus viruses and biological transmission of La Crosse virus by *Aedes triseriatus*. Am J Trop Med Hyg 1972;21:476-81.
16. Reed LJ, Muench HA. A simple method of estimating fifty per cent endpoints. Am J Trop Med Hyg 1938;27:493-7.
17. Cully JF Jr, Heard PB, Wesson DM, Craig GB Jr. Antibodies to La Crosse virus in eastern chipmunks in Indiana near an *Aedes albopictus* population. J Am Mosq Control Assoc 1991;7:651-3.
18. Grimstad PR, Barrett CL, Humphrey RL, Sinsko MJ. Serologic evidence for widespread infection with La Crosse and St. Louis encephalitis viruses in the Indiana human population. Am J Epidemiol 1984;119:913-30.
19. Hawley WA, Pumpuni CB, Brady RH, Craig GB Jr. Overwintering survival of *Aedes albopictus* (Diptera: Culicidae) eggs in Indiana. J Med Entomol 1989;26:122-9.
20. Nawrocki SJ, Hawley WA. Estimation of the northern limits of distribution of *Aedes albopictus* in North America. J Am Mosq Control Assoc 1987;3:314-7.
21. Hanson SM, Craig GB Jr. *Aedes albopictus* (Diptera: Culicidae) eggs: field survivorship during northern Indiana winters. J Med Entomol 1995;32:599-604.
